# Increased expression of pAKT is associated with radiation resistance in cervical cancer

**DOI:** 10.1038/sj.bjc.6603180

**Published:** 2006-05-23

**Authors:** T-J Kim, J-W Lee, S Y Song, J-J Choi, C H Choi, B-G Kim, J-H Lee, D-S Bae

**Affiliations:** 1 Department of Obstetrics and Gynecology, Samsung Medical Center, Sungkyunkwan University School of Medicine, 50 Ilwon-Dong, Gangnam-gu, Seoul 135-710, Korea; 2 Department of Pathology, Samsung Medical Center, Sungkyunkwan University School of Medicine, 50 Ilwon-Dong, Gangnam-gu, Seoul 135-710, Korea

**Keywords:** cervical neoplasms, radiation resistance, pAKT, PI3K, immunohistochemistry

## Abstract

Phosphorylated AKT (pAKT) is a major contributor to radioresistance in human cancers. The aim of this study was to investigate the association of pAKT expression and radiation resistance in cervical cancer. A retrospective review was made of the records of 27 women who received primary radiation therapy due to locally advanced cervical cancer (LACC) with FIGO stage IIB–IVA. Nine patients regarded as radiation resistant developed local recurrences with a median progression free interval of 9 months. Eighteen patients did not show local recurrences, and were regarded as a radiation-sensitive group. Using pretreatment paraffin-embedded tissues, we evaluated pAKT expression by immunohistochemistry. A significant association was found between the level of pAKT expression and local recurrence. Immunohistochemical staining for pAKT was significantly more frequent in the radiation-resistant than in the radiation-sensitive group (*P*=0.004). The mean progression-free survival was 86 months for patients with pAKT-negative staining (19 cases) and 44 months for patients with pAKT-positive expression (eight cases) (*P*=0.008). These results suggest that signalling from phosphatidylinositide 3-kinase/pAKT can lead to radiation resistance, and that evaluation of pAKT may be a prognostic marker for response to radiotherapy in LACC.

Cervical cancer is still the second most common cancer in women worldwide, despite the existence of effective screening methods (Waggoner, 2003). Radiation therapy can be used to treat all stages of cervical cancer, but in early-stage disease, it is usually reserved for medically unfit patients. Including adjuvant radiotherapy following surgery, which is used in approximately ⅓ of patients with stage IB disease, radiotherapy is the most commonly used treatment modality for >60% of cases of cervical cancer (Chung *et al*, 2005). The most significant change in the standard radiation treatment of cervical cancer has been cisplatin-containing concurrent chemotherapy with radiation for patients with locoregionally advanced disease (Keys *et al*, 1999; Morris *et al*, 1999; Rose *et al*, 1999). Despite the fact that concurrent chemoradiation demonstrated a marked improvement in pelvic disease control and survival, the resistance of tumour cells to radiation remains a major therapeutic problem. Although tumour size and international federation of gynaecology and obstetrics (FIGO) stage may serve successfully as markers for responsiveness to radiotherapy, they are not likely to fully account for the observed variability. New markers are needed to predict more accurately the response to radiotherapy of an individual patient.

The phosphatidylinositide 3-kinase (PI3K)/AKT-signalling pathway has recently been demonstrated to be a major survival signal in cancer cells. Phosphatidylinositide 3-kinase/AKT pathway has epidermal growth factor receptor (EGFR) and insulin-like growth factor signalling as upstreams, and it crosstalks with RAS/MAP kinase pathway. Indeed, the role of this pathway in tumorigenesis has been extensively investigated and altered expression or mutations of many components of this pathway have been implicated in human cancer (Vivanco and Sawyers, 2002). Phosphorylated and activated AKT leads to inhibition of apoptosis by inactivating several proapoptotic proteins, including BAD, BAX, and caspase 9, and also by inducing the expression of antiapoptotic proteins such as BCL2, BCLXL, FLIP, cIAP2, XIAP, and survivins (Mitsiades *et al*, 2002). Recent studies have shown that the PI3K/AKT cell survival pathway is activated post ionising radiation and UV radiation. Phosphatidylinositide 3-kinase/AKT-mediated survival pathways may fight imminent cell death and possibly induce insensitivity of tumour cells to radiation therapy (Zhan and Han, 2004). The increased expression of pAKT has been linked to decreased radiation responsiveness in various cancers, including head and neck squamous cell carcinoma, lung carcinoma, glioblastoma, and prostate and breast cancer (Gupta *et al*, 2002; Blackhall *et al*, 2003; Choe *et al*, 2003; Dent *et al*, 2003; Gupta *et al*, 2003; Liao *et al*, 2003; Vijapurkar *et al*, 2003; Schmitz *et al*, 2004). Therefore, the status of AKT may serve as a prognostic marker and the inhibition of PI3K/AKT-signalling pathway may provide an additional targeted approach to improve the outcome of radiotherapy.

With respect to cervical cancer, there have been a few studies about PI3K/AKT-signalling and related pathways. Epidermal growth factor receptor expression is higher in cervical intraepithelial neoplasia and cervical cancers than in normal controls (Mathur *et al*, 2000). Overexpression of the EGFR is associated with a poorer prognosis in patients with cervical cancer (Kersemaekers *et al*, 1999). Preclinical studies have shown that radiotherapy with a combination of antibodies to EGFR can improve local tumour control (Pillai *et al*, 1998; Nasu *et al*, 2001). Epidermal growth factor receptor signal functions through the activation of AKT. Phosphoinositide-3-kinase catalytic alpha, involved in the PI3K/AKT-signalling pathway, is known to be an oncogene associated with cervical cancer (Ma *et al*, 2000). In addition, the PI3K catalytic subunit is amplified in cervical cancer, and the inhibition of PI3K by LY294002 radiosensitises human cervical cancer cell lines (Lee *et al*, 2006). Therefore, we thought it would be worthwhile to investigate the components of PI3K/AKT pathway in cervical cancer.

In this study, we evaluate activated AKT using immunohistochemistry in patients with locally advanced cervical cancer (LACC), and have correlated pAKT staining with local recurrence following radiation in the patients studied. We suggest that activated AKT may lead to radiation resistance, and that PI3K/pAKT signalling may constitute a therapeutic target in cervical cancer.

## MATERIALS AND METHODS

### Patient selection

Between 1994 and 2001, a total of 119 patients with LACC stage IIB–IVA were treated by primary radiotherapy in the Department of Radiation Oncology, Samsung Medical Center, Sungkyunkwan University School of Medicine. From among them, 19 cases (15.9%) of pelvic recurrence were identified. Owing to the limited availability of tissue block specimens, we could not use 10 tissue blocks among 19 cases with local recurrence: the tissue biopsy was performed at outside clinics in five cases; and the biopsy was performed at out institution but tissue blocks were not available in four cases; and the size of the tissue block was too small to study in one case. We tried to match cases and controls (nine cases and 18 controls). Therefore, we studied 27 patients whose preradiation cervical biopsy specimens were available. Study records were reviewed according to institutional review board guidelines. Slides were prepared from the paraffin-embedded tissue blocks from these 27 patients. Included were 18 patients who showed no local recurrence until at least 3 years after treatment, except for one patient who died 14 months after pelvic radiotherapy due to paraaortic lymph node recurrence without evidence of pelvic disease. This comprised the radiation-sensitive group. The remaining nine cases with punch biopsy specimen availability came from the group of 19 who had pelvic recurrence, the radiation-resistant group.

We measured the tumour volume on MRI. The tumour volume was defined as volume calculated by the following formula: 0.524 × largest anteroposterior dimension × largest width dimension × largest craniocaudal dimension (Campbell *et al*, 1982).

All of these patients were given external beam radiotherapy (EBRT) as well as high-dose rate (HDR) intracavitary brachytherapy (Lee *et al*, 2004; Nakano *et al*, 2005). The whole pelvis total dose was 5040 Gy with 1.8 Gy of daily fraction, administered five times a week. High-dose rate brachytherapy was started 4–5 weeks after the initiation of the EBRT. The dose of HDR brachytherapy was 24 Gy at point A, with 4 Gy per fraction twice a week for 3 weeks.

### Immunohistochemical staining

Immunohistochemical staining was performed under the standard, avidin–biotin complex–peroxidase method (DakoCytomation, Denmark) using formalin-fixed, paraffin-embedded tissue sections. Four-*μ*m-thick tissue sections were mounted on poly-L-lysine-coated glass slides and dried at 37.8°C overnight. The sections were deparaffinised in xylene, washed in graded ethanol, and finally washed in phosphate-buffered saline (pH 7.4). To increase specificity and sensitivity, samples were pretreated with target retrieval citrate buffer (pH 6.0, S 2367; DakoCytomation, Denmark), in a microwave for 20 min. The endogenous peroxidase activity was blocked with 3% H_2_O_2_ for 15 min and the samples were preincubated with a protein-blocking solution for 10 min. Slides were stained with the IHC-specific phosphorylated Ser 473 Akt antibody (1:40 dilution, Cell Signalling TECHNOLOGY® Inc., Beverly, MA, USA) at 4°C overnight in humid chamber, as described by Zhou *et al* (2000). Slides were washed three times in phosphate-buffered saline and then incubated with a biotinylated goat anti-rabbit secondary antibody for 30 min at room temperature. Antigen–antibody complexes were detected with the avidin–biotin complex–peroxidase method, using diamminobenzidine as a chromogen substrate (Vectastain ABC-kit, Vector Laboratories, Burlingame, CA, USA), according to the manufacturer's protocol. Tissue sections were lightly counterstained with haematoxylin and then examined by light microscopy. One dedicated gynaecologic pathologist (SYS) and one gynaecologic oncologist (JWL) reviewed the blinded slides and evaluated the immunohistochemical data, independently. The agreement between the two reviewers was very strong. The staining was scored on a scale from 0 to 3+ as follows: 0, no staining; 1+, <50% with weak intensity; 2+, more than 50% with weak or moderate intensity; 3+, more than 50% with strong intensity. For statistical analysis of survival curve, we classified the pAKT expression of score 0 or 1+ as pAKT negative, score 2+ or 3+ as pAKT positive. Because keratinised tumour cells often caused artefactual staining, these keratinised areas were excluded from the analysis (Gupta *et al*, 2002).

### Statistical analysis

Statistical calculations were carried out using SPSS for Windows version 13.0 (SPSS Inc., Chicago, IL, USA). For continuous variables, the Mann–Whitney *U*-test was used. For categorical variables, linear by linear association was performed. To evaluate the relationship of pAKT staining to progression-free survival (PFS), we calculated Kaplan–Meier curves in conjunction with the log-rank test.

## RESULTS

Patient characteristics are described in Table 1. The median follow-up for the patients studied was 54 months. At the time of analysis, eight patients had died. One of eight died 96 months after radiotherapy, of causes unrelated to cervical cancer. One patient in the radiation-sensitive group developed paraaortic lymph node recurrence 10 months after pelvic radiotherapy and died 4 months later. There were no significant differences in age, cell type, histologic grade, tumour size, overall duration of therapy, and FIGO stage between the two groups.

Immunohistochemical expression of pAKT was examined in 27 cervical squamous cell carcinomas (18 in the radiation-sensitive and nine in the radiation-resistant group). The results are summarised in Table 2. There was no strong staining (3+) of pAKT in the radiation-sensitive group. There was no case without pAKT staining in the radiation-resistant group. Expression of pAKT was significantly more frequent in the radiation-resistant group, compared to the radiation-sensitive group (*P*=0.004).

**Figure 1:**
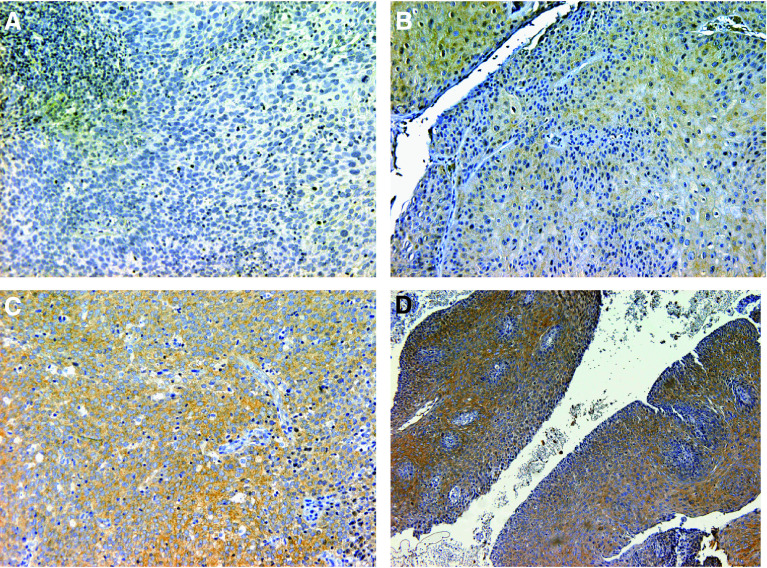
Representative examples of pAKT staining in cervical cancer. (**A**) No staining; (**B**) <50% with weak intensity (1+); (**C**) more than 50% with weak or moderate intensity (2+); (**D**) more than 50% with strong intensity (3+) (**A**–**C**, × 200; **D**, × 100).

Figure 1 illustrates the pAKT staining. It should be noted that false-positive staining was noted on keratin both in keratin pearls and in the epithelium, but this staining was excluded from evaluation.

**Figure 2:**
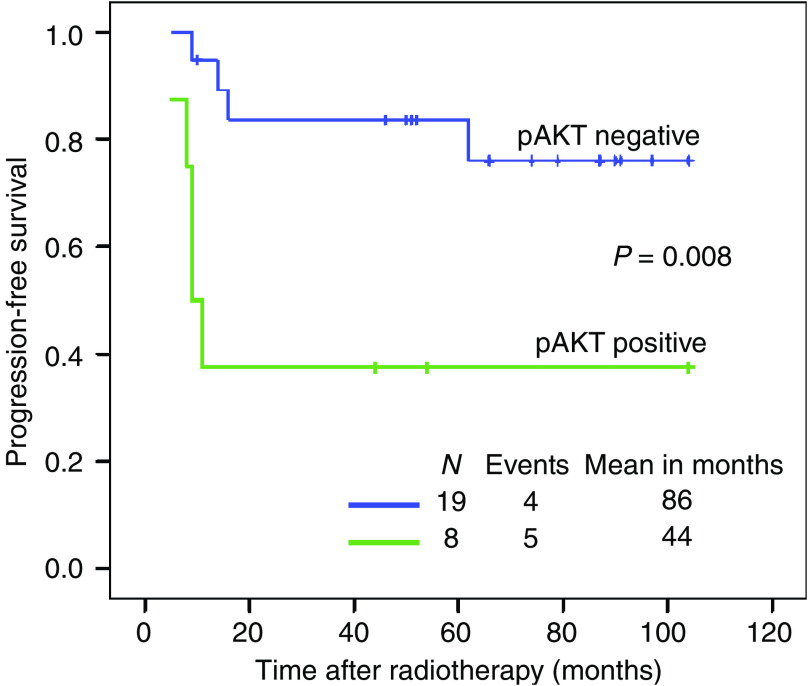
Univariate progression-free survival (PFS) analysis of pAKT expression in 27 cases of locally advanced cervical cancer. The ‘pAKT positive’ expression in pretreatment biopsies is a poor prognostic factor for PFS (*P*=0.008).

The PFS of all the patients studied was analysed based upon pAKT expression. The mean PFS was 86 for patients with pAKT-negative staining and 44 months for patients with pAKT-positive expression. The patients with pAKT-positive expression showed the poorer prognosis (*P*=0.008) (Figure 2).

## DISCUSSION

The results of this study evaluating histological pAKT expression prior to treatment in cervical cancer patients treated with primary radiotherapy show a significant association between pAKT expression and local recurrence. In addition, univariate survival analysis showed pAKT-positive tissue was associated with poor PFS following radiotherapy.

There is only one other study of AKT expression in cervical cancer treated with radiotherapy (Lee *et al*, 2005). Interestingly, however, Lee *et al* suggest the opposite conclusion: decreased pAKT correlated with increased disease progression, although it was difficult to determine whether this finding is based on pretreatment patient samples. Both studies utilised immunohistochemical staining using the same antibody. The difference between the two seems to be the way to interpret the data. We cannot explain these discrepant results, exactly. Nonetheless, we made a discussion on the results from the Lee's paper on the following points: The finding that diminished pAKT expression was significant for diminished overall survival (OS) is not a common characteristic of pAKT in human cancer. Also, their conclusion that only increased HER2 expression associated with improved OS is quite an opposite conclusion to the previous paper (Nakano *et al*, 1997). In addition, Lee suggested that ‘PI3K inhibition radiosensitises cervix cancer’ in his recent publication (Lee *et al*, 2006).

Radiation produces highly reactive free radicals, such as reactive oxygen species, which in turn interact with DNA to produce strand breaks that interfere with the cell's ability to reproduce. These molecules also function as intracellular messengers involved in the production of cytokines, growth factors, gene transcription, and apoptosis (Sen and Packer, 1996; Lander, 1997). Activated AKT can be induced by radiation, and it also may decrease imminent cell death or apoptosis from radiation injury. The mechanisms by which PI3K/AKT signalling functions in radiation responses may include regulation of mitochondrial proteins, transcription factors, translation machinery, and cell-cycle progression (Testa and Bellacosa, 2001; Zhan and Han, 2004).

There are three types of AKT (AKT-1, -2, and -3) in human tissues. However, the exact role of each has not been established in cervical cancer. Owing to the advent of high-quality phosphorylated pan-AKT serine 473 antibodies, it is possible to measure the phosphorylation of all three AKT family members using immunohistochemistry to examine AKT activity (Malik *et al*, 2002). We also evaluated all three AKT using phosphorylated pan-AKT antibody.

Clinical staging fails to detect extension of disease to the paraaortic lymph nodes in approximately 17% of patients with stage IIB cervical carcinoma, and in 29% of those with stage III disease. Such patients have ‘geographic’ treatment failure if standard radiation therapy ports are used (Hacker, 1988). Since we did not perform routine paraaortic sampling nor extended-field radiation in patients with cervical cancer, it is appropriate to define patients with local failure after primary radiation therapy, but not with distant failure only, as the ‘radiation-resistant’ group.

To compare PFS according to pAKT staining, we grouped cancer tissues with no or 1+ staining into ‘pAKT-negative’, and 2+ or 3+ staining into ‘pAKT-positive’. The pattern of staining was mostly cytoplasmic. Since the epithelial layer in some normal cervical tissues showed weak cytoplasmic staining for pAKT, it is appropriate to group both no staining and 1+ staining into ‘pAKT negative’.

We reviewed the clinical factors relevant to radiation in four pAKT-negative, radiation-resistant patients. Although all had low-stage diseases (stage IIB), they tended to be older in age, have greater tumour volume, higher grade, and shorter overall treatment duration than five pAKT-positive, radiation-resistant patients (Table 3). However, all these data showed no statistical significance.

The present retrospective study has a few limitations. Because of the low rate of cervical cancer with local recurrence after radiotherapy and a rarity of pretreatment tissue, the number of patients studied was small. Therefore, it is not easy to generalise these results into the larger population of patients with cervical cancer due to selection bias. We could not but limit the association of the pAKT expression with cervical carcinoma as descriptive rather than drawing a definitive conclusion based on statistical analyses. We hope that our observation from this small series would potentially prompt large, multi-institutional investigations. Small sample size also made it difficult to perform multivariate analyses in order to evaluate the pAKT expression as a predictor for radiation response. A criticism of this study would be its reliance on a single readout of Akt pathway activation. An additional demonstration of frequent phosphorylation of other pathway components, such as the downstream effectors mTOR and FOXO, or downregulation of phosphatase and tensin, will be needed.

In this study, we investigated whether pAKT expression measured in biopsy specimens of pretreatment human cervical cancers is associated with the response to radiation. If the PI3K/AKT pathway mediates radiation resistance in cervical cancer, then examination of activity of this pathway might predict the recurrence rate in patients with cervical cancer treated similarly with primary radiation. We found pretreatment pAKT expression to be significantly associated with local control of the cancer and with PFS. To more clearly evaluate the predictive role of pAKT to the radiation response in patients with cervical cancer, further studies with larger sample size will be performed.

## Figures and Tables

**Table 1:** Patient characteristics

**Characteristics**	**Radiation sensitive (*n*=18)**	**Radiation resistant (*n*=9)**	***P*-value**
Median age, years (range)	65 (38–80)	59 (36–73)	0.410
			
*Stage*			0.633
IIB	11 (61%)	5(56%)	
IIIB	7 (39%)	3 (33%)	
IVA		1 (11%)	
			
*Cell type*			1.00
Squamous cell	18 (100%)	9 (100%)	
			
*Histologic grade*			0.198
I	6 (33%)	1 (11%)	
II	10 (56%)	6 (67%)	
III	2 (11%)	2 (22%)	
			
Median tumour volume, cm^3^ (range)	19.1 (0.79–89.9)	31.4 (7.86–81.9)	0.777
Median OTT, days (range)	54.5 (41–77)	56 (50–74)	0.290
Median PFS, months (range)	70 (10–104)	9.0 (5–62)	<0.001
			
*Recurrence*			
Local	0	9	
Distant	1	1^a^	
			
*Current status*			
NED	16	0	
AWD	0	3	
DOD	1	6	
DWOD	1	0	

OTT: overall treatment time; PFS: progression-free survival; NED: no evidence of disease; AWD: alive with disease; DOD: dead of disease; DWOD: dead without disease.

a One patient had local and paraaortic lymph node recurrence at the same time.

**Table 2:** Results of immunohistochemical analysis for pAKT in radiation-sensitive and radiation-resistant cervical cancer tissues

	**pAKT staining**				
	**Negative (*n*=19)**		**Positive (*n*=8)**		
	**0**	**1+**	**2+**	**3+**	**Total (*N*=27)**
Radiation-sensitive	7	8	3	0	18
Radiation-resistant	0	4	2	3	9

The expression of pAKT was significantly more frequent in radiation-resistant than radiation-sensitive tissues (*P=0.004*).

**Table 3:** Clinical factors of four pAKT-negative radiation-resistant patients

	**Stage**	**Age**	**Grade**	**TV (cm^3^)**	**OTT (days)**
Case no. 1	IIB	59	II	51.9	58
Case no. 2	IIB	47	III	18.9	50
Case no. 3	IIB	73	III	31.4	52
Case no. 4	IIB	73	II	81.8	54
Median		66		41.7	53

TV: tumour volume; OTT: overall treatment time.
